# Hurricane Michael and Adverse Birth Outcomes in the Florida Panhandle: Analysis of Vital Statistics Data

**DOI:** 10.1017/dmp.2021.367

**Published:** 2022-03-03

**Authors:** Emily W. Harville, Ke Pan, Leslie Beitsch, Samendra P. Sherchan, Elaina Gonsoroski, Christopher Uejio, Maureen Y. Lichtveld

**Affiliations:** 1 Department of Epidemiology, School of Public Health and Tropical Medicine, Tulane University, New Orleans, LA, USA; 2 Department of Behavioral Sciences and Social Medicine, College of Medicine, Florida State University, Tallahassee, FL, USA; 3 Department of Environmental Health Sciences, School of Public Health and Tropical Medicine, Tulane University, New Orleans, LA, USA; 4 Department of Geography, College of Social Sciences and Public Policy, Florida State University, Tallahassee, FL, USA; 5 Department of Environmental and Occupational Health, Graduate School of Public Health, University of Pittsburgh, Pittsburgh, PA, USA

**Keywords:** birth weight, disaster, natural, fetal growth, preterm birth, trimester

## Abstract

**Objective:**

The aim of this study was to examine birth outcomes in areas affected by Hurricane Michael.

**Methods:**

Vital statistics data of 2017–2019 were obtained from the state of Florida. Births occurring in the year before and after the date of Hurricane Michael (October 7, 2018) were used. Florida counties were divided into 3 categories reflecting extent of impact from Hurricane Michael. Birth outcomes including incidence of preterm birth (PTB), low birth weight (LBW), and small for gestational age (SGA) were also compared before and after Hurricane Michael. Spontaneous and indicated PTBs were distinguished based on previously published algorithms. Multiple regression was used to control for potential confounders.

**Results:**

Both LBW (aRR 1.19, 95% CI: 1.07, 1.32) and SGA (aRR 1.11, 95% CI: 1.01, 1.21) were higher in the year after Michael than the year before in the most-affected area; a similar effect was not seen in other areas. A stronger effect was seen for exposure in the first trimester or in the 2 months after Michael than in the second or third trimester.

**Conclusion:**

Consistent with many previous studies, this study of Hurricane Michael found an effect on fetal growth.

Disasters have been associated with poor birth outcomes. Most consistent are modest effects on birth weight, generally examined by region of residence and timing of birth. Magnitude of effect has ranged from 160 g decrement in birth weight associated with the Wenchuan and Haiti earthquakes^1,2^ down to single-digit associations with wildfires,^3^ landmine explosions,^4^ with other examinations of earthquakes^5–7^ and major disaster declarations^8^ finding effect estimates that fall in between. (However, other studies have found no effects of hurricanes on birth weight.^9–11^) Effects on preterm birth (PTB)/gestational age have been found much less often.^12^ Studies that have addressed both birth weight and gestational age have tended to find stronger effects on birth weight, if they find an effect at all,^7,13–28^ and only a few have found an effect on PTB but not low birth weight (LBW; birth weight < 2500 g).^29,30^

This is surprising as LBW and PTB co-occur, with early delivery being a common cause of LBW. Best practice is usually considered to analyze small for gestational age (SGA; usually defined as birth weight < 10th percentile for gestational age) rather than LBW per se, because birth weight is affected both by length of gestation and fetal growth. PTB and LBW have overlapping but not completely identical risk factors^31,32^; for instance, male fetuses are usually larger but are more likely to be born early, and smoking is a clear risk factor for LBW but is less strongly associated with PTB. If disaster has different effects on these outcomes, it may indicate mechanisms; for instance, effects limited to growth-related outcomes might suggest a focus on behavior, for example, nutrition and smoking.

A secondary issue is that of timing of exposure to disaster, where results have again been inconsistent for both birth weight and gestational age. Studies of disaster and related time-specific stressors such as terrorist attacks have found no difference by trimester of exposure^13,18^; strongest effects in the first trimester^4–6,33,34^; strongest effects in the second trimester^3,14,21,23,24,35,36^; strongest effects in the third trimester^3,25^; as well as variation in strongest effects by exposure, outcome,^5^ and their combinations.^8^ Theoretically, effects in the first trimester may be more strongly related to placentation, while later effects are more likely to relate to growth or immediate labor triggers.

In examining effects of disaster on pregnant women, it may therefore be useful to compare and contrast effects on birth weight, weight for gestational age, and gestational age. Unlike other hurricanes where the focus has been effects on major cities,^26,37^ Hurricane Michael hit a primarily rural, less populated area. This study analyzed the data for changes in birth outcomes to address 3 questions: (1) Was Hurricane Michael associated with changes in incidence of LBW and PTB? (2) Did the storm have differential associations depending on its timing in pregnancy?_—_and (3) Were there similar associations with LBW and PTB, and if not, can subtypes of these conditions be distinguished, perhaps to provide information on distinctions between the two?

## Methods

### Data Source

Vital statistics data of 2017–2019 were obtained from the state of Florida. Births occurring in the year before and after the date of Hurricane Michael (before: October 6, 2017–October 6, 2018; after: October 7, 2018–October 7, 2019) were used to assess changes in births and birth outcomes in counties affected by Hurricane Michael.

### Affected areas

Based on FEMA disaster declarations,^38^ Florida counties were divided into 3 categories reflecting extent of impact from Hurricane Michael: counties receiving both public and individual assistance (Area A), counties receiving only public assistance (Area B), and counties receiving neither public nor individual assistance (Area C). (Individual assistance is provided to individuals who have sustained losses, although it does not compensate for all losses caused by disaster, while public assistance funds repair or reconstruction of public facilities or infrastructure.) The category of each county can be found in Supplementary Table 1. The hypothesis underlying such exposure categorization is that increasing extent of impact is associated with an increased proportion of the population having severe exposure, an increased average exposure, and increased exposure to secondary traumas (devastated neighborhoods, community member deaths) among those not directly affected. Women were classified as exposed based on their residential address.

### Outcomes

The total number of births during October 6, 2017–October 6, 2018 and those during October 7, 2018–October 7, 2019 were compared. Birth outcomes including incidence of preterm birth (PTB), low birth weight (LBW), and small for gestational age (SGA) were also compared before and after Hurricane Michael. PTB was defined as a birth before 37 weeks of gestation. LBW was defined as a birth weight of an infant of 2500 g or less, regardless of gestational age. SGA was defined by birth weight below the 10th percentile for gestational age based on the national standard.^39^

We used the algorithm reported by Klebanoff et al. to distinguish spontaneous versus indicated preterm births.^40^ Indicators of spontaneous births included premature rupture of membranes, labor characteristics, and vaginal birth, while induction and C-section were associated with indicated PTB (see reference for full algorithm). Using the Ohio birth certificates from 2006 to 2012, the kappa statistic of the algorithm was 0.68 (95% CI: 0.52, 0.83); predictive values for spontaneous and indicated onset were 85% (95% CI: 75%, 92%) and 89% (95% CI: 71%, 98%). While generally a good-quality algorithm, distinguishing spontaneous and indicated births can be difficult even in medical records, especially in cases of premature rupture of membranes.^41^

### Timing of Exposure to Hurricane Michael

Births were categorized into 5 categories according to time relative to Hurricane Michael. Babies who were delivered before October 7, 2018 were categorized into category “before” Hurricane Michael. Women’s exposure was categorized by trimester on October 7, 2018: first trimester (< 14 weeks), second trimester (14 ≤ 28 weeks), and third trimester (28 weeks+), as well as pregnant within 2 months after Hurricane Michael in a “within 2 months after” category. The timing of 2 months was chosen because it allows those pregnancies to have more than 42 weeks (ie, to be complete) before October 6, 2019.

### Covariates

Maternal age, race, education, and whether enrolled in the U.S. Department of Agriculture’s special supplemental nutrition program for women, infants, and children (WIC) program were considered as confounders because those variables are known risk factors for adverse birth outcomes and their distribution among women giving birth could have shifted after the hurricane. Access to antenatal care (ANC) services before and after Hurricane Michael was evaluated by whether pregnant women had any ANC visit before delivery, the month of the first ANC visit, and the Kotelchuck Index. There are 4 adequacy categories in the Kotelchuck Index: adequate plus, adequate, intermediate, and inadequate.^42^ The 4-level categorization is important as it helps distinguish complicated pregnancy (which may require extra prenatal visits) from those that receive limited or acceptable levels of care.

### Statistical Analyses

Covariate missing data were minimal: maternal age (0.0025%), education (0.97%), race/ethnicity (1.28%), pre-pregnancy body mass index (BMI) (5.63%), whether enrolled in the WIC program (1.24%), smoking during pregnancy (0.38%), and alcohol drinking during pregnancy (1.51%)—so, a complete case analysis was used. Paired t-tests were used to compare the number of births before and after Hurricane Michael. Linear regression was used to assess whether the change in the number of births was different across different areas. Log-binomial regression was used for binary outcomes; if these failed to converge, logistic models were used. All of the estimates were compared unadjusted and after adjusting for potential confounders. Comparisons were made in 2 directions: before and after Hurricane Michael, and among different levels of exposure. In order to assess these factors jointly, an interaction term was also added in unadjusted and adjusted models. The analysis was also performed stratified by trimester of exposure. Statistical analyses were performed using the software SAS 9.4 (SAS Inc., Cary, NC).

These analyses were conducted under a waiver of informed consent and approved by the Institutional Review Boards of Tulane University, Florida State University, and the Florida Department of Health.

## Results

There was a total of 218 903 and 217 966 live births in Florida between October 6, 2017–October 6, 2018 and October 7, 2018– October 7, 2019, respectively. The total numbers of live births were not significantly different before and after Hurricane Michael within each affected area. The change in the number of births across categories of affected areas was not significantly different (P for interaction = 0.83).

The racial composition, mean maternal age, pre-pregnancy BMI, and gestational age at birth were similar before and after Hurricane Michael within each affected area (Table 1). The percentage of women who were enrolled in the WIC program decreased after Hurricane Michael in all areas. In all areas, the maternal education distribution changed after Hurricane Michael so that a smaller proportion of women giving birth were in the middle educational category (more than a high school but less than a college degree) compared to before.

### Low Birth Weight

There was a higher proportion of LBW among counties in area A after Michael, adjusting for age, education, ethnicity, pre-pregnancy BMI, and participation in WIC (aRR = 1.19, 95% CI: 1.07, 1.32), but the proportion of LBW did not change after Michael among areas B and C (Table 2). Mean decline in birth weight was 4.7 g overall, 29.5 g in area A, 27.8 g in area B, and 3.4 g in area C. The change in the proportion of LBW after Michael was greater in area A compared to area C (aRR = 1.19, 95% CI: 1.07–1.32 vs aRR = 1.003, 95% CI: 0.98–1.02, *P* for interaction = 0.002). However, the effect was not different between areas B and C.

### Preterm Birth

There was a higher proportion of PTB within area B after Michael, adjusting for covariates (aRR=1.16, 95%CI 1.04, 1.29), but the proportion of PTB did not change after Michael within area A or C (Table 2). There was an interaction between exposure to Michael and area, in that the proportion of PTB was greater in area B compared to area C, but the effect was not different between area A and C.

### Small for Gestational Age

There was a higher proportion of SGA in area A after Michael, adjusting for covariates (aRR = 1.11, 95% CI: 1.02, 1.21) (see Table 2). Compared to area C, the effects of Michael on the proportion of SGA were different in area A (*P* for interaction = 0.01) but not in area B (*P*= 0.40).

### Timing of Exposure

#### Low birth weight

Within the most affected area A, exposure during the first trimester (aOR = 1.25, 95% CI: 1.03–1.51) or pregnancy within 2 months after Michael (aOR = 1.36, 95% CI: 1.12–1.66) showed the highest increases compared to before the hurricane. These increases were not seen in the unaffected areas; the moderately affected area showed a small increase in risk among conceptions in the 2 months after the hurricane (Table 3). Comparing across regions, the most affected area A was at similar risk to areas B and C for births prior to the hurricane (area A vs area C: aOR = 0.901, 95% CI: 0.84–1.00; area B vs area C: aOR = 0.98, 95% CI: 0.87–1.10) (Table S3).

#### Preterm birth

Within the moderately affected area B, exposure during the first trimester (aOR = 1.22, 95% CI: 1.00–1.50) or pregnancy within 2 months after Michael (aOR = 1.20, 95% CI: 0.99–1.50) showed the highest increase in PTB compared to before the hurricane. These increases were not seen in the area A or C (see Table 3). When compared across regions, incidence of PTB was lower in area A for exposure in the second trimester (aOR = 0.85, 95% CI: 0.73–0.99).

#### Small for gestational age

Within the most affected area A, exposure within 2 months after Michael (aOR = 1.29, 95% CI: 1.09–1.53) showed increased risk for SGA compared to before the hurricane, which was not seen in the unaffected areas (see Table 3). Women living in the most affected area A were at small increased risk of delivering an SGA child compared to those in the unaffected area C, regardless of timing (Table S3).

### Spontaneous and Indicated PTB

Area B seemed to be at higher risk for induced PTB (Table 4), regardless of trimester of exposure (Table S3), and before the storm had been at lower risk of spontaneous PTB (aOR = 0.75, 95% CI: 0.64–0.87). Area A was at lower risk of induced PTB after exposure in the second trimester (aOR = 0.81, 95% CI: 0.65–0.99). Overall incidence of spontaneous PTB in areas A and B was higher for conceptions in the 2 months after the storm, but this may have been a random variation (aOR = 1.13, 95% CI: 0.90–1.41 for area A; aOR = 1.10, 95% CI: 0.82–1.48 for area B; Table S3).

## Discussion

### Principal Findings

The impact of a hurricane on a less populated area on maternal and child health outcomes, specifically LBW, PTB, and SGA was examined. Vital statistics data from counties were categorized by degree of damage caused by Hurricane Michael. Overall, an increase in LBW and SGA was found in the most-affected areas, consistent with previous studies,^43^ and with similar effect sizes.^2,20,25^ These effects appeared to be stronger among women who experienced the storm in the first trimester, or who conceived shortly after the hurricane, compared to those exposed in the second or third trimester. These findings are consistent with several studies showing the strongest effects with first-trimester exposure.^4–6,33,34^

### Limitations

Strengths include the large sample size and consideration of multiple definitions of exposure and outcome. Limitations of the study include the reliance on vital statistics, defining exposure by county-level damage and timing, and the lack of information on physiologic, behavioral, or social mechanisms of effect. Future analyses will explore some of these topics in more detail. While a validated classification system was used for spontaneous versus medically indicated preterm births, such systems are still limited relative to detailed research or medical record review. Further subtyping was not possible, and distinctions among preterm premature rupture of membranes (PPROM) and preterm labor or different indications for induction (such as pre-eclampsia or fetal growth restriction) might be relevant to understanding effects of a complex exposure like disaster. A few studies have examined effects of disaster on PPROM alone,^5,44,45^ and 1 recent analysis examined only spontaneous births, whether preterm or not,^46^ but that has not necessarily led to any more consistent results.

### Interpretation

Few studies have considered the post-disaster period. One study of 9/11 found minimal difference in effects between women who were pregnant at the time of the attack and those who became pregnant later,^47^ while another found decreased odds of moderate PTB for several weeks post-disaster and increased odds of very LBW around 4 months later,^14^ but no effects on other outcomes or during other post-disaster time periods. A study of Hurricane Katrina suggested some effects lingered 5–7 years later,^48^ while 1 study of county-level PTB rates after disasters found effects lasting only for 2 to 3 weeks after the hurricane exposure.^46^ As many of the effects of disaster exposure_—_stress, economic problems, lack of access to health care_—_linger for months or years, it is reasonable that exposure to post-disaster life early in pregnancy would have similar effects.

Also consistent with previous studies is the lack of effect on PTB in the most-exposed counties,^7,12–28^ especially confusing as there was an increase in the moderately exposed counties. This is somewhat surprising, as PTB and LBW often go hand-in-hand and some large and detailed studies have found an effect.^8,46^ Gestational age is often measured less precisely and consistently than birth weight, which may make effects more difficult to detect. For birth weight, where an effect was found, the absolute size of the change was small. Third-trimester effects must be examined carefully, as women who give birth preterm have a shorter third trimester, but this should not affect estimate of first-trimester effects. One analysis of the spatial hazards data across the United States concluded that disaster affected birth weight more strongly in counties that were less vulnerable, while gestational age was most affected in more vulnerable counties,^8^ and Sun et al. also concluded that effects on PTB are stronger in areas with high social vulnerability.^46^ Based on the Centers for Disease Control and Prevention social vulnerability index,^49^ the counties in the affected areas were either ranked highly (Holmes, Washington, Jackson, Calhoun, Liberty, Franklin, Gadsden, Taylor) or moderate to highly vulnerable (Leon, Gulf, Bay, Wakulla). However, a stronger effect was found on birth weight compared to PTB. It is possible that a hurricane could have specific effects, triggering preterm labor alone, for instance, or lead to complications such as hypertensive disorders that would be mainly apparent in induced PTB, but examination of spontaneous versus induced PTB failed to reveal any patterns.

There are several possible mechanisms by which disaster could affect birth weight, including health behaviors such as increased smoking and an unhealthy diet, reduced fetal growth due to higher stress hormones and blood pressure, and increased susceptibility to infection. Our models controlled for covariates that appeared to change between time periods, but the possibility for residual confounding remains (although there is no obvious candidate that would vary across regions, the effects are small enough that relatively minor imbalances could cause a difference). Unlike Hurricane Katrina,^11^ the overall number of births in the affected areas did not change significantly; therefore, selective fertility or migration is unlikely to be a cause of the findings.^50^

The decreased enrollment in WIC also suggests possible nutritional effects. Although enrollment is theoretically possible during and after disaster, there are several possible reasons for this decline: physical and communication barriers; evacuation separating women from normal care; the general mental toll of rebuilding (including clean-up, dealing with insurance, and taking care of family and neighbors), which may limit the time and energy available to sign up. Facility shutdowns are another consideration. In some of the affected counties, such as Leon, there was no interruption to services, and benefits were uploaded to recipients’ accounts so they did not need to come into the office. In others, such as Bay County, health departments and offices were shut down for some weeks.

Our analytical strategy, which is at the individual level and examines maternal residence at the time of the hurricane, is probably the type most frequently used in the literature and allows for control of individual-level confounding. Defining exposure by place of delivery might provide additional information on effects on the health care system, but maternal residence provides a better estimate of individual overall hurricane exposure. Other analysis strategies have been used, such as comparisons of county-level rates^46,51^ or means,^8^ time series,^14^ sibling studies,^4^ and treating the hurricane as a time-varying exposures in a proportional hazards model.^52^ Difference-in-difference analysis assumes confounders are time-invariant, which may not be realistic, as disaster may induce differential shifts in covariates such as ethnic distribution.^6,51^ Studies also vary in whether the control group is other unaffected areas or the same area, cohort, or clinical population in a previous or later time period; both types of comparisons were performed. Studies with very fine time scales^46,52^ or that allow only for exposure in late pregnancy^52^ inherently focus on hurricane exposure as a short-term trigger. Given the long-term effects of disaster on so many aspects of life, this seems to represent a limited window.

## Conclusion

This study adds to the body of evidence, suggesting an effect of disaster on pregnancy outcomes, particularly fetal growth, and that effects early in pregnancy may be particularly severe. The lack of effect on preterm birth is also consistent with many previous studies and warrants further study as to why, if stress is a cause of PTB,^53^ PTB so rarely rises in the aftermath of a major stressor. Some argue for PTB as an adaptive response under some conditions; the inverse_—_adaptive extension of gestation under conditions of stress_—_could also be hypothesized.^54^ It may be that disaster-associated stress contributes to PTB more in the context of chronic or social determinant of health-associated stress, rather than as a short-term stressor. Further studies employing more detailed phenotyping and measurement of mediating factors may be necessary to more comprehensively ascertain these disaster-related effects on maternal and child health (MCH). However, given the large amount of research on the topic, it may be time to move to developing interventions to improve post-disaster outcomes.

## Supplementary Material

1

## Figures and Tables

**Table 1.** Description of study population, Florida vital statistics, 2017–2019

	FEMA individual^a^ (Area A)			FEMA public (Area B)			not affected (Area C)		
		
	Before	After		Before	After		Before	After	
					
	Mean (SE)/ N (%)		p-value	Mean (SE)/ N (%)		p-value	Mean (SE)/ N (%)		p-value

**Total number of births**	7555	7261	0.492	4334	4406	0.213	207014	206299	0.417

**Maternal age**	27.73 (5.72)	27.86 (5.75)	0.157	27.92 (5.89)	28.19 (5.63)	0.024	29.18 (5.83)	29.33 (5.85)	<0.0001

**Maternal education level**									

High School or GED or less	3173 (42.67%)	3109 (43.83%)	0.001	1858 (43.35%)	1980 (45.30%)	0.015	86579 (42.26%)	86503 (42.27%)	<0.0001

Some College Credit, but No Degree or Associate Degree	2370 (31.87%)	2069 (29.17%)		1412 (32.94%)	1314 (30.06%)		59307 (28.95%)	57797 (28.24%)	

Bachelor’s Degree and above	1893 (25.46%)	1915 (27.00%)		1016 (23.71%)	1077 (24.64%)		58962 (28.78%)	60360 (29.49%)	

**Maternal ethnicity**									

Non-Hispanic White	4455 (59.55%)	4191 (58.51%)	0.003	2955 (68.29%)	2925 (66.64%)	0.298	83808 (41.16%)	83183 (40.71%)	<0.0001

Hispanic white	387 (5.17%)	450 (6.28%)		498 (11.51%)	526 (11.98%)		61018 (29.97%)	62365 (30.52%)	

Black	2206 (29.49%)	2169 (30.28%)		552 (12.76%)	613 (13.97%)		45250 (22.22%)	44801 (21.92%)	

Other	433 (5.79%)	353 (4.93%)		323 (7.44%)	325 (7.40%)		13526 (6.64%)	13998 (6.85%)	

**WIC program**	3772 (50.98%)	3346 (46.51%)	<0.0001	1762 (41.62%)	1735 (39.54%)	0.050	91147 (44.50%)	85758 (42.14%)	<0.0001

**Pre-pregnancy BMI**	27.81 (7.31)	27.86 (7.38)	0.702	27.07 (6.76)	27.32 (7.02)	0.096	26.76 (6.50)	26.95 (6.60)	<0.0001

**Smoking during pregnancy**	534 (7.10%)	597 (8.28%)	0.007	445 (10.29%)	506 (11.50%)	0.070	8126 (3.94%)	7755 (3.77%)	0.004

FEMA, Federal Emergency Management Agency; GED, General Educational Development; WIC, Women’s, Infant’s, and Children; BMI, body mass index

a Individual-level FEMA aid is available in the most-damaged areas; less-damaged areas are eligible only for public assistance.

**Table 2.** Changes in perinatal outcomes after Hurricane Michael among different areas, Florida vital statistics, 2017–2019

		Unadjusted model			Adjusted model^a^		
	
		FEMA individual (Area A)	FEMA public (Area B)	not affected (Area C)	FEMA individual^&^ (Area A)	FEMA public (Area B)	not affected (Area C)

**Low birth weight (LBW)**	**N**	1230/14097	693/8508	32335/398486	1230/14097	693/8508	32335/398486

	**RR (95%CI)**	1.210 (1.087, 1.347)	1.125 (0.975, 1.298)	1.000 (0.979, 1.021)	1.190 (1.070, 1.323)	1.095 (0.950, 1.262)	1.003 (0.982, 1.024)

	**p-value** ^b^	0.001	0.109	–	0.002	0.228	–

**Preterm birth (PTB)**	**N**	1639/13265	1069/8141	47621/379087	1639/13265	1069/8141	47621/379087

	**RR (95%CI)**	0.976 (0.891, 1.069)	1.182 (1.057, 1.323)	1.012 (0.995, 1.029)	0.958 (0.876, 1.049)	1.157 (1.034, 1.294)	1.010 (0.993, 1.027)

	**p-value** ^#^	0.441	0.007	–	0.264	0.018	–

**Small for gestational age (SGA)**	**N**	1691/13265	808/8141	41949/379066	1691/13265	808/8141	41949/379066

	**RR (95%CI)**	1.136 (1.039, 1.242)	1.055 (0.925, 1.202)	0.976 (0.959, 0.994)	1.111 (1.018, 1.213)	1.043 0.916, 1.187)	0.986 (0.968, 1.004)

	**p-value** ^#^	0.001	0.254	–	0.009	0.399	–

RR, relative risk; FEMA, Federal Emergency Management Agency; BMI, body mass index; WIC, Women’s, Infants, and Children’s

a LBW adjusting for: mother’s education, age, ethnicity, smoking during pregnancy, and whether in WIC program; PTB, SGA adjusting for: mother’s age, education, ethnicity, pre-pregnancy BMI, smoking during pregnancy, and whether in WIC program

b RR compares the year after Hurricane Michael to the year before; p-value is for the interaction between effect of disaster and area

**Table 3.** Perinatal outcomes by timing of pregnancy relative to Hurricane Michael within each area

	Unadjusted			Adjusting^a^ for maternal characteristics		
	
	OR	95%CI		OR	95%CI	

**Low birth weight (LBW)**						

*Area A*^b^ (N=1230/14097)						

3^rd^ trimester vs before^c^	0.891	0.721	1.100	0.884	0.715	1.094

2^nd^ trimester vs before	1.156	0.965	1.385	1.145	0.955	1.374

1^st^ trimester vs before	1.270	1.052	1.532	1.247	1.032	1.507

Within 2 months after vs before	1.397	1.150	1.698	1.361	1.118	1.656

*Area B* (N=693/8508)						

3^rd^ trimester vs before	0.686	0.504	0.936	0.65	0.483	0.900

2^nd^ trimester vs before	0.949	0.739	1.22	0.917	0.712	1.181

1^st^ trimester vs before	1.106	0.861	1.421	1.085	0.843	1.398

Within 2 months after vs before	1.322	1.022	1.711	1.293	0.998	1.677

*Area C* (N=32335/398486)						

3^rd^ trimester vs before	0.645	0.618	0.674	0.645	0.617	0.674

2^nd^ trimester vs before	1.005	0.970	1.041	1.006	0.971	1.043

1^st^ trimester vs before	0.979	0.944	1.016	0.994	0.957	1.031

Within 2 months after vs before	1.013	0.972	1.056	1.017	0.975	1.060

**Preterm birth (PTB)**						

*Area A* (N=1639/13265)						

3^rd^ trimester vs before	0.567	0.462	0.696	0.557	0.453	0.684

2^nd^ trimester vs before	0.888	0.754	1.046	0.878	0.745	1.035

1^st^ trimester vs before	1.036	0.876	1.225	1.013	0.856	1.199

Within 2 months after vs before	1.116	0.937	1.328	1.080	0.906	1.288

*Area B* (N=1069/8141)						

3^rd^ trimester vs before	0.591	0.451	0.775	0.578	0.441	0.759

2^nd^ trimester vs before	1.161	0.952	1.417	1.132	0.927	1.382

1^st^ trimester vs before	1.241	1.013	1.521	1.223	0.997	1.500

Within 2 months after vs before	1.233	0.989	1.539	1.203	0.963	1.502

*Area C* (N=47621/379087)						

3^rd^ trimester vs before	0.591	0.569	0.614	0.588	0.567	0.611

2^nd^ trimester vs before	1.050	1.019	1.081	1.046	1.015	1.077

1^st^ trimester vs before	1.008	0.977	1.039	1.015	0.983	1.047

Within 2 months after vs before	0.982	0.948	1.017	0.981	0.947	1.017

**Small for gestational age (SGA)**						

*Area A* (N=1691/13265)						

3^rd^ trimester vs before	1.141	0.967	1.347	1.135	0.959	1.342

2^nd^ trimester vs before	1.089	0.931	1.275	1.067	0.910	1.251

1^st^ trimester vs before	1.090	0.921	1.29	1.063	0.896	1.261

Within 2 months after vs before	1.332	1.124	1.577	1.291	1.087	1.533

*Area B* (N=808/8141)						

3^rd^ trimester vs before	1.003	0.783	1.286	0.98	0.762	1.259

2^nd^ trimester vs before	0.975	0.774	1.227	0.951	0.754	1.200

1^st^ trimester vs before	1.194	0.953	1.496	1.184	0.942	1.487

Within 2 months after vs before	1.097	0.853	1.411	1.090	0.845	1.405

*Area C* (N=41949/379066)						

3^rd^ trimester vs before	0.968	0.936	1.001	0.971	0.939	1.004

2^nd^ trimester vs before	0.985	0.955	1.016	0.994	0.963	1.026

1^st^ trimester vs before	0.969	0.938	1.001	0.991	0.959	1.025

Within 2 months after vs before	0.958	0.923	0.995	0.971	0.935	1.008

a LBW adjusting for: mother’s education, age, ethnicity, smoking during pregnancy, and whether in WIC program; PTB, SGA adjusting for: mother’s age, education, ethnicity, pre-pregnancy BMI, smoking during pregnancy, and whether in WIC program

b Before: gave birth before Hurricane Michael

c Area A=FEMA individual; Area B=FEMA public; Area C=non-affected

**Table 4.** Changes in induced preterm birth and spontaneous preterm birth after Hurricane Michael among different areas

		Unadjusted model			Adjusted model^a^		
	
		FEMA individual (Area A)	FEMA public (Area B)	not affected (Area C)	FEMA individual^&^ (Area A)	FEMA public (Area B)	not affected (Area C)

**Induced preterm birth**	**N**	815/12441	657/7729	23671/355137	815/12441	657/7729	23671/355137

	**RR (95%CI)**	1.003 (0.878, 1.145)	1.156 (0.998, 1.339)	1.024 (0.999, 1.050)	0.986 (0.864, 1.125)	1.116 (0.965, 1.291)	1.015 (0.991, 1.040)

	**p for interaction** ^b^	0.758	0.111	–	0.673	0.206	–

**Spontaneous preterm birth**	**N**	822/12448	408/7480	23737/355203	822/12448	408/7480	23737/355203

	**RR (95%CI)**	0.951 (0.834, 1.086)	1.279 (1.057, 1.548)	0.997 (0.972, 1.021)	0.931 (0.817, 1.062)	1.254 (1.037, 1.517)	1.002 (0.978, 1.027)

	**p for interaction** ^b^	0.499	0.011	–	0.285	0.021	–

a Adjusting for: mother’s age, education, ethnicity, pre-pregnancy BMI, smoking during pregnancy, and whether in WIC program

b RR compares the year after Hurricane Michael to the year before; p-value is for the interaction between effect of disaster and area

## Abbreviations:

ANC: antenatal care
BMI: body mass index
FEMA: Federal Emergency Management Agency
LBW: low birth weight
OR: odds ratio
PTB: preterm birth
SGA: small for gestational age
