# Supplementary material for: Hurricane Michael and Adverse Birth Outcomes in the Florida Panhandle: Analysis of Vital Statistics Data
Source: Disaster Med Public Health Prep. Author manuscript; Available in PMC 2023 Feb 4. (PMC9440161; doi:10.1017/dmp.2021.367)
Supplement: 1 [file NIHMS1766407-supplement-1.docx]

Table S1. Categorization of counties by Hurricane Michael FEMA aid eligibility

| BAY | Individual and public (area A) |
| --- | --- |
| CALHOUN | Individual and public (area A) |
| FRANKLIN | Individual and public (area A) |
| GADSDEN | Individual and public (area A) |
| GULF | Individual and public (area A) |
| HOLMES | Individual and public (area A) |
| JACKSON | Individual and public (area A) |
| LEON | Individual and public (area A) |
| LIBERTY | Individual and public (area A) |
| TAYLOR | Individual and public (area A) |
| WAKULLA | Individual and public (area A) |
| WASHINGTON | Individual and public (area A) |
| HAMILTON | public (area B) |
| JEFFERSON | public (area B) |
| MADISON | public (area B) |
| SUWANNEE | public (area B) |
| OKALOOSA | public (area B) |
| WALTON | public (area B) |
| COLUMBIA | no aid (area C) |
| DIXIE | no aid (area C) |
| ESCAMBIA | no aid (area C) |
| GILCHRIST | no aid (area C) |
| LAFAYETTE | no aid (area C) |
| ALACHUA | no aid (area C) |
| SANTA ROSA | no aid (area C) |
| BAKER | no aid (area C) |
| BRADFORD | no aid (area C) |
| BREVARD | no aid (area C) |
| BROWARD | no aid (area C) |
| CHARLOTTE | no aid (area C) |
| CITRUS | no aid (area C) |
| CLAY | no aid (area C) |
| COLLIER | no aid (area C) |
| DESOTO | no aid (area C) |
| DUVAL | no aid (area C) |
| FLAGLER | no aid (area C) |
| HENDRY | no aid (area C) |
| HERNANDO | no aid (area C) |
| HIGHLAND | no aid (area C) |
| HILLSBOROUGH | no aid (area C) |
| INDIAN RIVER | no aid (area C) |
| LAKE | no aid (area C) |
| LEE | no aid (area C) |
| LEVY | no aid (area C) |
| MANATEE | no aid (area C) |
| MARION | no aid (area C) |
| MARTIN | no aid (area C) |
| MIAMI-DADE | no aid (area C) |
| MONROE | no aid (area C) |
| NASSAU | no aid (area C) |
| OKEECHOBEE | no aid (area C) |
| ORANGE | no aid (area C) |
| OSCEOLA | no aid (area C) |
| PALM BEACH | no aid (area C) |
| PASCO | no aid (area C) |
| PINELLAS | no aid (area C) |
| POLK | no aid (area C) |
| PUTNAM | no aid (area C) |
| SARASOTA | no aid (area C) |
| SEMINOLE | no aid (area C) |
| ST JOHNS | no aid (area C) |
| ST LUCIE | no aid (area C) |
| SUMTER | no aid (area C) |
| UNION | no aid (area C) |
| VOLUSIA | no aid (area C) |

For a map, see the FEMA disaster declaration, https://www.fema.gov/disaster/4399

Table S2. Difference of perinatal outcomes by timing of pregnancy relative to Hurricane Michael among different areas

|  | **Unadjusted** | | |  | **Adjusting for maternal characteristics^a^** | | |
| --- | --- | --- | --- | --- | --- | --- | --- |
|  | **OR** | **95%CI** | |  | **OR** | **95%CI** | |
| **Low birth weight (LBW)** | | | | | | | |
| *Before^b^* | | | | | | | |
| Area A vs C^c^ | 0.970 | 0.889 | 1.059 |  | 0.914 | 0.837 | 0.998 |
| Area B vs C | 0.941 | 0.839 | 1.056 |  | 0.982 | 0.874 | 1.103 |
| *3^rd^ trimester* | | | | | | | |
| Area A vs C | 1.339 | 1.099 | 1.631 |  | 1.254 | 1.028 | 1.530 |
| Area B vs C | 1.001 | 0.749 | 1.339 |  | 1.004 | 0.749 | 1.346 |
| *2^nd^ trimester* | | | | | | | |
| Area A vs C | 1.116 | 0.949 | 1.312 |  | 1.04 | 0.883 | 1.225 |
| Area B vs C | 0.889 | 0.710 | 1.114 |  | 0.895 | 0.713 | 1.124 |
| *1^st^ trimester* | | | | | | | |
| Area A vs C | 1.258 | 1.061 | 1.491 |  | 1.147 | 0.966 | 1.362 |
| Area B vs C | 1.063 | 0.848 | 1.333 |  | 1.073 | 0.854 | 1.347 |
| *Within 2 months after* | | | | | | | |
| Area A vs C | 1.339 | 1.120 | 1.601 |  | 1.224 | 1.021 | 1.466 |
| Area B vs C | 1.229 | 0.973 | 1.553 |  | 1.250 | 0.987 | 1.582 |
| **Preterm birth (PTB)** | | | | | | | |
| *Before* | | | | | | | |
| Area A vs C | 1.004 | 0.932 | 1.081 |  | 1.014 | 0.941 | 1.093 |
| Area B vs C | 0.957 | 0.869 | 1.054 |  | 1.040 | 0.943 | 1.146 |
| *3^rd^ trimester* | | | | | | | |
| Area A vs C | 0.962 | 0.793 | 1.169 |  | 0.960 | 0.790 | 1.166 |
| Area B vs C | 0.957 | 0.742 | 1.235 |  | 1.022 | 0.791 | 1.320 |
| *2^nd^ trimester* | | | | | | | |
| Area A vs C | 0.849 | 0.731 | 0.985 |  | 0.852 | 0.734 | 0.989 |
| Area B vs C | 1.058 | 0.888 | 1.262 |  | 1.126 | 0.943 | 1.344 |
| *1^st^ trimester* | | | | | | | |
| Area A vs C | 1.032 | 0.885 | 1.203 |  | 1.012 | 0.868 | 1.181 |
| Area B vs C | 1.179 | 0.983 | 1.413 |  | 1.254 | 1.045 | 1.505 |
| *Within 2 months after* | | | | | | | |
| Area A vs C | 1.140 | 0.969 | 1.340 |  | 1.116 | 0.948 | 1.314 |
| Area B vs C | 1.202 | 0.982 | 1.471 |  | 1.275 | 1.041 | 1.562 |
| **Small for gestational age (SGA)** | | | | | | | |
| *Before* | | | | | | | |
| Area A vs C | 1.073 | 0.994 | 1.157 |  | 1.027 | 0.951 | 1.110 |
| Area B vs C | 0.844 | 0.759 | 0.939 |  | 0.872 | 0.783 | 0.971 |
| *3^rd^ trimester* | | | | | | | |
| Area A vs C | 1.265 | 1.088 | 1.471 |  | 1.201 | 1.030 | 1.399 |
| Area B vs C | 0.876 | 0.698 | 1.099 |  | 0.880 | 0.699 | 1.107 |
| *2^nd^ trimester* | | | | | | | |
| Area A vs C | 1.186 | 1.030 | 1.366 |  | 1.102 | 0.955 | 1.272 |
| Area B vs C | 0.836 | 0.679 | 1.028 |  | 0.834 | 0.677 | 1.028 |
| *1^st^ trimester* | | | | | | | |
| Area A vs C | 1.207 | 1.034 | 1.407 |  | 1.102 | 0.942 | 1.288 |
| Area B vs C | 1.041 | 0.851 | 1.273 |  | 1.041 | 0.849 | 1.277 |
| *Within 2 months after* | | | | | | | |
| Area A vs C | 1.491 | 1.276 | 1.743 |  | 1.366 | 1.166 | 1.600 |
| Area B vs C | 0.967 | 0.767 | 1.218 |  | 0.979 | 0.775 | 1.237 |

^a^LBW adjusting for: mother's education, age, ethnicity and whether in WIC program; PTB, SGA, C-section, breastfeeding adjusting for: mother's age, education, ethnicity, pre-pregnancy BMI and whether in WIC program

^b^Before: gave birth before Hurricane Michael

^c^Area A=FEMA individual; Area B=FEMA public; Area C=non-affected

Table S3. Spontaneous vs. induced preterm birth by timing of pregnancy relative to Hurricane Michael among different areas

|  | **Unadjusted** | | |  | **Adjusting for maternal characteristics^a^** | | |
| --- | --- | --- | --- | --- | --- | --- | --- |
|  | **OR** | **95%CI** | |  | **OR** | **95%CI** | |
| **Induced preterm birth (IPTB)** | | | | | | | |
| *Before* | | | | | | | |
| Area A vs C | 0.993 | 0.896 | 1.101 |  | 0.999 | 0.901 | 1.108 |
| Area B vs C | 1.215 | 1.078 | 1.369 |  | 1.345 | 1.193 | 1.517 |
| *3^rd^ trimester* | | | | | | | |
| Area A vs C | 1.049 | 0.805 | 1.368 |  | 1.044 | 0.800 | 1.363 |
| Area B vs C | 1.210 | 0.874 | 1.675 |  | 1.312 | 0.946 | 1.82 |
| *2^nd^ trimester* | | | | | | | |
| Area A vs C | 0.799 | 0.648 | 0.984 |  | 0.806 | 0.653 | 0.994 |
| Area B vs C | 1.283 | 1.031 | 1.598 |  | 1.390 | 1.114 | 1.734 |
| *1^st^ trimester* | | | | | | | |
| Area A vs C | 1.003 | 0.811 | 1.241 |  | 0.997 | 0.805 | 1.235 |
| Area B vs C | 1.470 | 1.175 | 1.840 |  | 1.590 | 1.269 | 1.993 |
| *Within 2 months after* | | | | | | | |
| Area A vs C | 1.135 | 0.911 | 1.415 |  | 1.122 | 0.898 | 1.400 |
| Area B vs C | 1.369 | 1.057 | 1.773 |  | 1.477 | 1.138 | 1.916 |
| **Spontaneous preterm birth (SPTB)** | | | | | | | |
| *Before* | | | | | | | |
| Area A vs C | 1.015 | 0.918 | 1.123 |  | 1.025 | 0.926 | 1.135 |
| Area B vs C | 0.697 | 0.598 | 0.812 |  | 0.745 | 0.639 | 0.868 |
| *3^rd^ trimester* | | | | | | | |
| Area A vs C | 0.888 | 0.675 | 1.167 |  | 0.883 | 0.671 | 1.162 |
| Area B vs C | 0.732 | 0.494 | 1.085 |  | 0.773 | 0.521 | 1.146 |
| *2^nd^ trimester* | | | | | | | |
| Area A vs C | 0.905 | 0.741 | 1.105 |  | 0.896 | 0.733 | 1.095 |
| Area B vs C | 0.820 | 0.624 | 1.076 |  | 0.850 | 0.647 | 1.117 |
| *1^st^ trimester* | | | | | | | |
| Area A vs C | 1.076 | 0.875 | 1.323 |  | 1.039 | 0.844 | 1.279 |
| Area B vs C | 0.903 | 0.682 | 1.195 |  | 0.947 | 0.714 | 1.255 |
| *Within 2 months after* | | | | | | | |
| Area A vs C | 1.167 | 0.935 | 1.456 |  | 1.128 | 0.902 | 1.409 |
| Area B vs C | 1.051 | 0.781 | 1.413 |  | 1.102 | 0.819 | 1.484 |

^a^Adjusting for: mother's age, education, ethnicity, pre-pregnancy BMI and whether in WIC program

Table S4. Induced preterm birth and spontaneous preterm birth by timing of pregnancy relative to Hurricane Michael within each area

|  | **Unadjusted** | | |  | **Adjusting^a^ for maternal characteristics** | | |
| --- | --- | --- | --- | --- | --- | --- | --- |
|  | **OR** | **95%CI** | |  | **OR** | **95%CI** | |
| **Induced preterm birth** | | | | | | | |
| *Area A* | | | | | | | |
| 3^rd^ trimester vs before | 0.600 | 0.454 | 0.793 |  | 0.589 | 0.445 | 0.779 |
| 2^nd^ trimester vs before | 0.863 | 0.686 | 1.085 |  | 0.855 | 0.679 | 1.077 |
| 1^st^ trimester vs before | 1.026 | 0.813 | 1.295 |  | 1.012 | 0.801 | 1.279 |
| Within 2 months after vs before | 1.147 | 0.904 | 1.456 |  | 1.116 | 0.878 | 1.418 |
| *Area B* | | | | | | | |
| 3^rd^ trimester vs before | 0.565 | 0.401 | 0.796 |  | 0.549 | 0.390 | 0.775 |
| 2^nd^ trimester vs before | 1.133 | 0.886 | 1.450 |  | 1.096 | 0.855 | 1.405 |
| 1^st^ trimester vs before | 1.229 | 0.957 | 1.579 |  | 1.199 | 0.932 | 1.543 |
| Within 2 months after vs before | 1.130 | 0.854 | 1.497 |  | 1.091 | 0.823 | 1.447 |
| *Area C* | | | | | | | |
| 3^rd^ trimester vs before | 0.568 | 0.538 | 0.599 |  | 0.563 | 0.534 | 0.594 |
| 2^nd^ trimester vs before | 1.073 | 1.031 | 1.117 |  | 1.061 | 1.019 | 1.105 |
| 1^st^ trimester vs before | 1.016 | 0.973 | 1.060 |  | 1.014 | 0.972 | 1.059 |
| Within 2 months after vs before | 1.003 | 0.956 | 1.053 |  | 0.994 | 0.947 | 1.043 |
| **Spontaneous preterm birth** | | | | | | | |
| *Area A* | | | | | | | |
| 3^rd^ trimester vs before | 0.538 | 0.404 | 0.717 |  | 0.529 | 0.396 | 0.705 |
| 2^nd^ trimester vs before | 0.916 | 0.735 | 1.142 |  | 0.901 | 0.722 | 1.123 |
| 1^st^ trimester vs before | 1.050 | 0.837 | 1.316 |  | 1.020 | 0.813 | 1.280 |
| Within 2 months after vs before | 1.091 | 0.859 | 1.385 |  | 1.052 | 0.827 | 1.336 |
| *Area B* | | | | | | | |
| 3^rd^ trimester vs before | 0.646 | 0.425 | 0.983 |  | 0.637 | 0.419 | 0.969 |
| 2^nd^ trimester vs before | 1.209 | 0.887 | 1.647 |  | 1.177 | 0.863 | 1.605 |
| 1^st^ trimester vs before | 1.284 | 0.935 | 1.762 |  | 1.280 | 0.931 | 1.758 |
| Within 2 months after vs before | 1.431 | 1.029 | 1.990 |  | 1.415 | 1.016 | 1.970 |
| *Area C* | | | | | | | |
| 3^rd^ trimester vs before | 0.615 | 0.585 | 0.648 |  | 0.614 | 0.583 | 0.647 |
| 2^nd^ trimester vs before | 1.028 | 0.987 | 1.070 |  | 1.031 | 0.990 | 1.073 |
| 1^st^ trimester vs before | 0.991 | 0.950 | 1.034 |  | 1.007 | 0.965 | 1.051 |
| Within 2 months after vs before | 0.949 | 0.904 | 0.997 |  | 0.956 | 0.910 | 1.004 |

^a^Adjusting for: mother's age, education, ethnicity, pre-pregnancy BMI and whether in WIC program
